# Modulation of the pupillary response by the content of visual working memory

**DOI:** 10.1073/pnas.1909959116

**Published:** 2019-10-21

**Authors:** Nahid Zokaei, Alexander G. Board, Sanjay G. Manohar, Anna C. Nobre

**Affiliations:** ^a^Oxford Centre for Human Brain Activity, Wellcome Centre for Integrative Neuroimaging, Department of Psychiatry, University of Oxford, OX3 7JX Oxford, United Kingdom;; ^b^Department of Experimental Psychology, University of Oxford, OX1 3UD Oxford, United Kingdom;; ^c^Nuffield Department of Clinical Neurosciences, University of Oxford, OX3 9DU Oxford, United Kingdom

**Keywords:** working memory, attention, pupillometry

## Abstract

Studies of selective attention during perception have revealed modulation of the pupillary response according to the brightness of task-relevant (attended) vs. -irrelevant (unattended) stimuli within a visual display. As a strong test of top-down modulation of the pupil response by selective attention, we asked whether changes in pupil diameter follow internal shifts of attention to memoranda of visual stimuli of different brightness maintained in working memory, in the absence of any visual stimulation. Across 3 studies, we reveal dilation of the pupil when participants orient attention to the memorandum of a dark grating relative to that of a bright grating. The effect occurs even when the attention-orienting cue is independent of stimulus brightness, and even when stimulus brightness is merely incidental and not required for the working-memory task of judging stimulus orientation. Furthermore, relative dilation and constriction of the pupil occurred dynamically and followed the changing temporal expectation that 1 or the other stimulus would be probed across the retention delay. The results provide surprising and consistent evidence that pupil responses are under top-down control by cognitive factors, even when there is no direct adaptive gain for such modulation, since no visual stimuli were presented or anticipated. The results also strengthen the view of sensory recruitment during working memory, suggesting even activation of sensory receptors. The thought-provoking corollary to our findings is that the pupils provide a reliable measure of what is in the focus of mind, thus giving a different meaning to old proverbs about the eyes being a window to the mind.

## Significance

We report, across 3 complementary studies, that attention in visual working memory leads to top-down modulations of the pupillary response, in the absence of any visual input or anticipation of any change in brightness. These findings strengthen the view of the sensory recruitment, all the way down to the earliest synapses in the visual system. The thought-provoking corollary to our findings is that the pupils provide a reliable measure of what is in the focus of the mind.

Working memory, our ability to hold in mind information for brief periods of time (1), is not a unitary phenomenon. Rather, current attempts to improve our understanding of this function consider working memory in terms of different states determined by the interaction between memory traces and attention (2–6). It has been shown that one can flexibly orient attention to different items in working memory, so that items can dynamically move in and out of the focus of attention (7, 8). Focusing attention on an item places it in a prioritized state to gate performance (9–12). The remaining items are still available for retrieval, but are considered to be in different representational state (7, 13, 14).

The attentional biases that operate during working-memory retention are closely linked to attentional processes at perception, with similar neural and behavioral markers for items in the focus of both internal and external attention (15). In perception, orienting attention involves preparing the sensory mechanisms, resulting in better and faster recall of the prioritized item with higher accuracy and fidelity. From a neural perspective, attended stimuli elicit a more vigorous and selective response in sensory regions (16). Interestingly, increasingly early modulations have been noted, all the way to the top-down modulation of the pupillary response to attended stimuli. More specifically, studies have shown that attending to bright stimuli induces a pupillary constriction relative to attending to dark stimuli, under identical visual input (17, 18).

Similarly, attention in working memory modulates sensory cortical responses to the prioritized memoranda in a way that is decodable during the working-memory retention period from the sensory regions and susceptible to disruptive transcranial magnetic stimulation (8, 19–21). Based on these findings, it has been proposed that attention can prioritize the sensory content of these mnemonic representations (5, 6). One important yet outstanding question, however, is whether directing attention to internal representations also involves reinstatement of sensory modulations all of the way down to the earliest synapses in the visual system, for example invoking pupillary responses when no differential light is presented nor is expected.

Recent studies have implicated the pupillary response in visual working memory, but not yet in a way that rules out modulation based on perceptual input or expectation of a change in brightness. Pupil responses have been reported to track selective encoding of items into visual working memory (22). When viewing an array of items with different brightness, the pupil size adapts to the brightness of the relevant items to be encoded, such that selective encoding of darker items results in an increase in pupil size (22). Such a finding is interesting, but is entirely explainable by the effects of spatial selective attention on the perceptual stimuli relevant for encoding (17, 18).

Modulation of pupil size has also been noted to track shifts of attention during the working-memory delay period (23). In this case, the screen was divided into a dark and a light half. The memory array consisted of 2 items presented on either side of a screen. The item relevant for performance became cued during the delay period, and the pupil size adjusted to the brightness of the relevant screen side. However, any change in pupil size may reflect shifts in gaze position to the attended location rather than a change in response to selective modulation of memory traces as it has recently been shown that shifts of attention are accompanied by gaze biases toward the spatial location of the attended memory item (24).

Another recent preprint (25) reports a study specially aimed at testing pupil responses linked to shifts of selective attention within working memory, but in this case it is not possible to separate attention-related modulation of working-memory memoranda from preparatory attention to anticipated probe stimuli. In this case, the brightness of the probe stimuli matched that of the cued memorandum. Thus, although suggestive evidence has not ruled out the possibility that pupil responses are modulated by attention to the sensory content of memorandum, no studies to date have provided unambiguous data to test this possibility.

Therefore, in the present study we examined top-down modulation of the pupillary response to memory representations in the absence of any brightness-related confounds that could lead to contaminations by perceptual attention or anticipation. The study was completed over 3 experiments. In the first experiment, participants kept 2 oriented gratings in mind, 1 bright and 1 dark. Fully predictive auditory retrospective cues presented during the working-memory delay (100% validity) indicated which memorandum to prioritize to guide performance. Importantly, regardless of whether the bright or dark grating was cued, the probe stimulus was identical, thus allowing us to test the role of top-down modulation of the pupillary response under identical visual input with no difference in anticipation of a bright or a dark probe. We hypothesized that prioritization of the darker grating during working-memory maintenance would elicit a dilation in pupil size compared to prioritizing bright gratings. The 2 subsequent experiments take a step further and ask whether modulation of the pupillary responses occurs even when brightness is an irrelevant feature of the prioritized stimulus. In these 2 experiments, prioritization of memory items was based on the spatial location of the grating either through retrospective auditory cues (Exp. 2) or by temporal expectations derived from learning that gratings at each spatial location were probed at different time points during the delay (Exp. 3). Finding pupillary modulation when stimulus brightness is entirely incidental and task-irrelevant would suggest some degree of integration among the various features in the memoranda relating to items in working memory, and obligatory retrieval of even irrelevant features of a recalled object. Thus, in addition to replicating the original study, the findings would also shed light on the nature of memory representations (26).

## Results

### Exp. 1: Orienting Attention to Bright vs. Dark Items in Visual Working Memory.

**Fig. 1.**
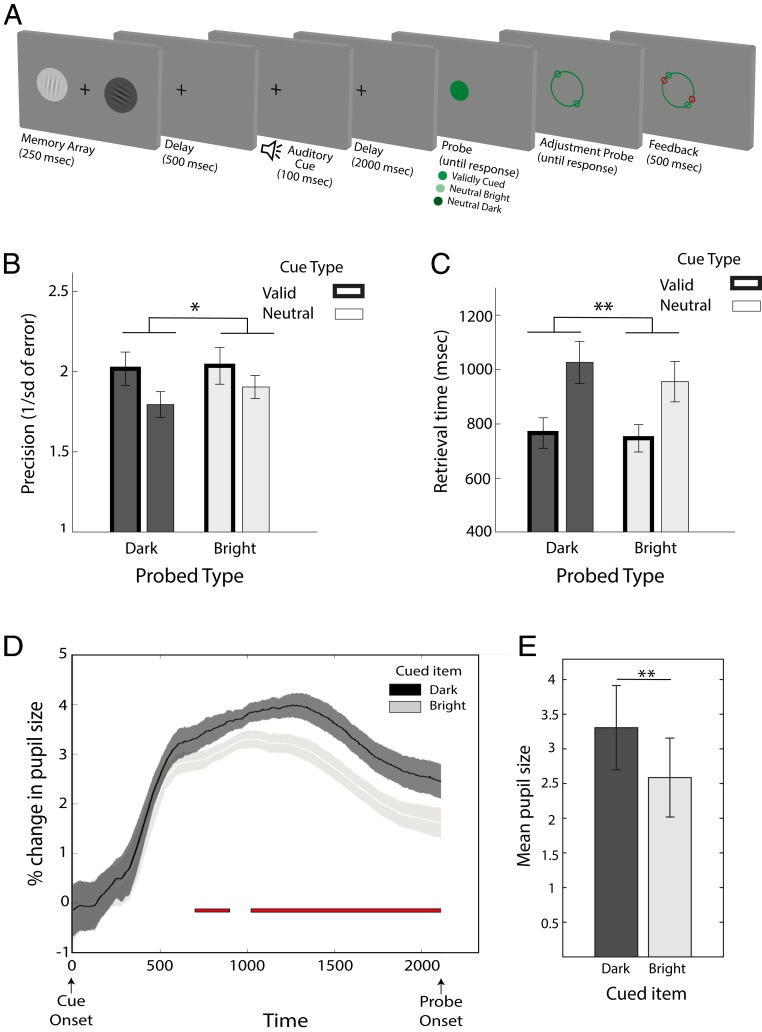
Exp. 1 task schematic and behavioral results. (*A*) During encoding, participants were presented with 2 randomly oriented gratings, in dark or bright gray, and were asked to keep in mind their orientations. During the memory delay, an informative auditory sound cued the participants to either the bright or dark grating (100% valid), or an uninformative auditory sound provided no information about the upcoming probe. This was followed by a delay before the presentation of the probe. For validly cued trials, the probe stimulus itself is not informative, and participants must rely on the information in the cue. They provide a full report by adjusting the response wheel to match the orientation of the relevant item and receive feedback. In trials with an uninformative neutral cue, the probe provides information about the item to be reported (light stimulus probes the light grating and dark stimulus probed the dark grating). Responses are then delivered in the same way as the validly cued trials. (*B*) Mean precision as a function of the probed item’s brightness (bright/dark) and cue type (valid/neutral). (*C*) Mean retrieval time as a function of probe item’s brightness and cue type. (*D*) The influence of cue on pupil diameter. Comparisons of these traces with each other are shown. Validly cued darker gratings elicited a larger change in pupil size compared to validly cued brighter gratings (red bar). The shaded area indicates the SE within subjects. (*E*) Mean pupil size from 500 ms after cue onset until probe was larger for trials in which the darker item was cued compared to trials in which the brighter item was cued. Error bars indicate SEM, calculated across participants. *P < 0.05 and **P < 0.005.

In Exp. 1, we used retrospective (retro-) cues to manipulate attention to items in visual working memory (Fig. 1*A*; see *Methods and Materials* for detailed description of the method). Participants viewed and encoded 2 oriented gratings, 1 bright and 1 dark, and were asked to keep in mind their orientations. On two-thirds of the trials, a valid auditory retrocue during the memory delay instructed participants about which item (bright or dark) should be used for reporting orientation when a probe item appeared. Importantly, the probe stimulus had a constant, intermediate level of brightness, and therefore could not itself induce any differential preparatory pupillary response according to cueing condition. On the remaining trials, a neutral auditory retrocue during the memory delay provided no information regarding the item to be probed. In such cases, the brightness of the probe indicated whether participants should reproduce the orientation of the bright or dark item.

#### Behavioral measures.

Repeated-measures ANOVAs tested the within-subjects effects of retrocue validity (valid vs. neutral) and brightness of the probed grating (dark vs. bright). We used working-memory precision as a measure of response accuracy, defined as the circular SD of response error, the difference between reported and veridical target angle (see *SI Appendix*, Fig. S1 for mixture modeling of response error). Responses for grating orientations were more precise in trials with valid compared to neutral retrocues (Fig. 1*B*) [main effect of validity, *F*(1, 21) = 5.8, *P* = 0.025, η^2^_p_ = 0.22]. There was no effect of brightness [main effect of brightness, *F*(1, 21) = 1.35, *P* = 0.26, η^2^_p_ = 0.06] or interaction between brightness and cue validity [*F*(1, 21) = 0.9, *P* = 0.37, η^2^_p_ = 0.04].

Retrieval times were calculated as the time between probe onset and the mouse click prior to the start of the reproduction. This measure of memorandum accessibility confirmed faster retrieval times for validly cued items [main effect of validity, *F*(1, 21) = 55.6, *P* < 0.001, η^2^_p_ = 0.55] (Fig. 1*C*). Brightness did not affect retrieval times [*F*(1, 21) = 5, *P* = 0.06, η^2^_p_ = 0.16] or interact with validity [*F*(1, 21) = 3.8, *P* = 0.065, η^2^_p_ = 0.15].

#### Pupil traces.

To test for top-down modulation of pupillary responses resulting from orienting attention to bright vs. dark items in working memory, we compared pupil size traces after the auditory retrocues (see *SI Appendix*, Fig. S2*A* for raw pupil trace for the whole trial duration). Time window of interest was between 500 ms after cue presentation and the onset of probe. Auditory sounds were counterbalanced between participants and, importantly, there was no significant difference in magnitude of the pupillary response to different sound cues, regardless of the condition, after correction for false-discovery rate (minimum *P* = 0.27).

We focused our pupil analysis on validly cued conditions only, for cued dark vs. cued bright gratings. The neutral cue condition was not included, as in this condition, participants held 2 items in working memory as opposed to only 1 in the validly cued condition. Therefore, the 2 conditions cannot be directly compared as any differences between the pupil size may reflect differences in the number of items held in working memory or in differential states of difficulty or arousal that this may generate.

To compare the pupil sizes of the 2 conditions of interest (cued dark vs. cued bright), we used permutation testing to avoid problems associated with multiple comparisons (27). In validly cued trials in which the darker grating was cued, there was a larger change in pupil size during memory delay (after 500 ms from cue offset) compared to trials in which the brighter grating was cued (Fig. 1*D*). This difference became significant 899 ms after the cue, until the probe.

To complement this analysis, we calculated the mean pupil size from 500 ms after the cue onset until the probe for trials in which the bright item or the dark item was cued. A paired sample *t* test revealed a significant difference between mean pupil size when dark versus bright item were cued [*t*(21) = 6.6, *P* < 0.001]. Darker items elicited a larger mean pupil size trace (Fig. 1*E*).

Thus, taken together, the results of Exp. 1 show that participants can orient attention on the basis of the brightness of a memorandum, resulting in improved recall precision and response times. Intriguingly, orienting attention to items of different brightness also results in top-down modulation of pupil size, even in the absence of any difference in brightness of the anticipated probe stimulus.

### Exp. 2: Pupillary Response According to Task-Irrelevant Brightness of Attended Stimuli.

**Fig. 2.**
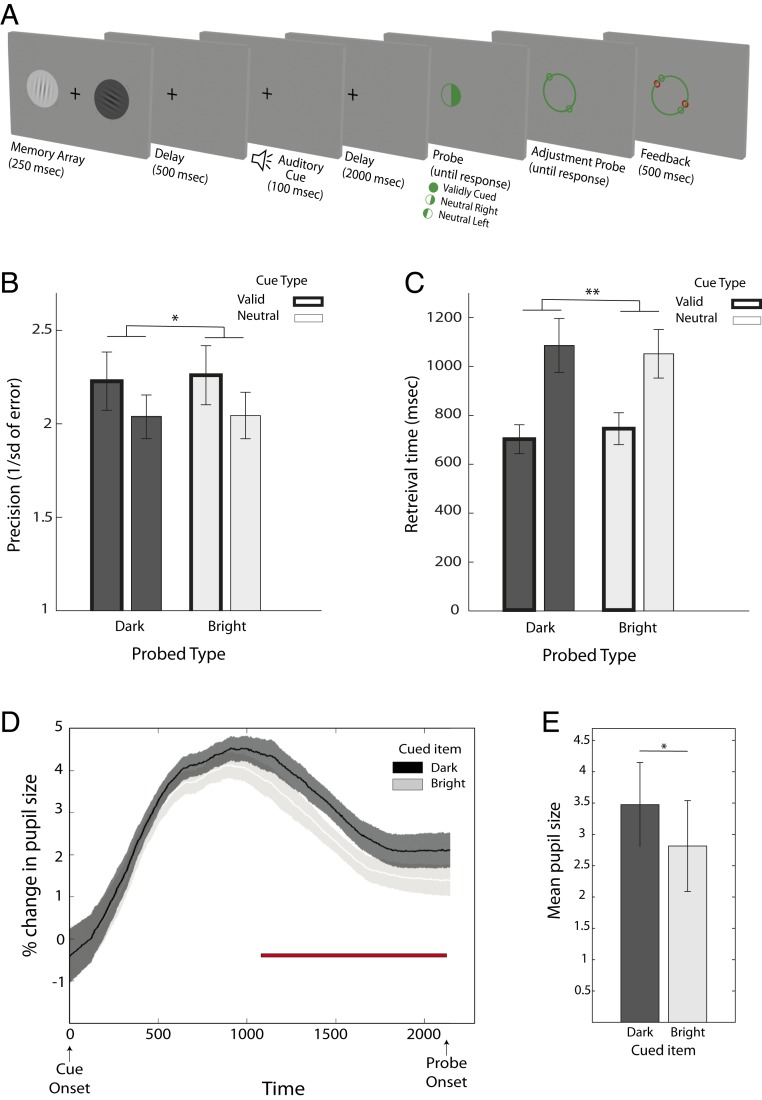
Exp. 2 task schematic and behavioral results. (*A*) Task was similar to Exp. 1, except for the meanings of the cues and the appearance of the probe stimuli. The cues either indicated the side of the to-be-probed item or was neutral. At probe, participants were presented with either a half-filled circle, indicating the spatial location of the grating that had to be recalled, or was a full circle in validly cued trials, ensuring that participants paid attention to the cue. (*B*) Mean precision as a function of the probed item’s side (bright/dark) and cue type (valid/neutral). (*C*) Mean retrieval time as a function of probe item’s side and cue type. (*D*) The influence of cue on pupil diameter. Comparisons of these traces with each other are shown. Validly cued darker gratings elicited a larger change in pupil size compared to validly cued brighter gratings. The shaded area indicates the SE within subjects. (*E*) Mean pupil size from 500 ms after cue onset until probe was larger for trials in which the darker item was cued compared to trials in which the brighter item was cued. Error bars indicate SEM, calculated across participants. *P < 0.05 and **P < 0.005.

In Exp. 2, we sought to replicate our results and go 1 step further in asking whether top-down modulation of pupil trace size occurred even when stimulus brightness was not the relevant feature for orienting attention or for reporting (as in Exp. 1), considering that the association between the sound cue and brightness could have been recalled in response to the cues. To this end, spatial retrocues were used to manipulate attention in working memory, and participants again reported grating orientation (Fig. 2*A*). The brightness of the attended vs. unattended memorandum was entirely incidental to the task.

#### Behavioral measures.

Repeated-measures ANOVAs tested the within-subject effects of cue validity (valid vs. neutral), side of the probed grating (right vs. left), and brightness of the probed item (dark vs. bright) (see *SI Appendix*, Fig. S1 for mixture modeling of response error). Precision measures showed reliable advantages of spatial relative to neutral retrocues (Fig. 2*B*) [a main effect of cue validity *F*(1, 22) = 7.8, *P* = 0.011, η^2^_p_ = 0.26]. There was no effect of side [*F*(1, 22) = 1.7, *P* = 0.2, η^2^_p_ = 0.06] or brightness of the probed item [*F*(1, 22) = 0.13, *P* = 0.72, η^2^_p_ = 0.009], and there were no interactions among any of the factors [cue × side: *F*(1, 22) = 0.102; cue × brightness: *F*(1, 22) = 0.029; brightness × side: *F*(1, 22) = 0.222; cue × side × brightness: *F*(1, 22) = 3.6].

Response times also revealed a significant benefit after valid compared to neutral retrocues [*F*(1, 22) = 26.02, *P* < 0.001, η^2^_p_ = 0.54] (Fig. 2*C*). Again, there was no effect of side [*F*(1, 22) = 4.1, *P* = 0.054, η^2^_p_ = 0.015], brightness [*F*(1, 22) = 1.8, *P* = 0.12], or interaction between any of the factors [*F*(1, 22) = 0.13, *P* = 0.72, η^2^_p_ = 0.002], and there were no interactions among any of the factors [cue × side: *F*(1, 22) = 0.05, η^2^_p_ = 0.001; cue × brightness: *F*(1, 22) = 0.09, η^2^_p_ = 0.003; brightness × side: *F*(1, 22) = 3.9, η^2^_p_ = 0.02; cue × side × brightness: *F*(1, 22) = 0.86, η^2^_p_ = 0.005].

#### Pupil traces.

To analyze the pupillary response according to the brightness of the cued item, we reorganized the trials in validly cued condition, based on the brightness of the cued item irrespective of its side (see *SI Appendix*, Fig. S2*B* for raw pupil trace for the whole trial duration). Time window of interest was between 500 ms after cue presentation and the onset of probe. This resulted in 2 conditions where either the bright or the dark grating was in the focus of attention. Comparisons were carried out using the method described for Exp. 1.

Trials in which the dark item was cued resulted in a larger pupillary response during working-memory retention than trials in which the bright item was cued (Fig. 2*D*). This difference remained significant from 1,065 ms after the onset of the auditory cue until the probe stimulus appeared.

As in Exp. 1, all auditory cues elicited a pupil response, but its magnitude was not affected by the different cue sounds (irrespective of cue condition, minimum *P* = 0.09). Furthermore, there was no significant difference in magnitude of the pupillary response between right versus left cued items at any time-point after correction for false-discovery rate (minimum *P* = 0.07).

To complement this analysis, we calculated mean pupil size from 500 ms after the cue onset until the probe for trials in which the bright item or the dark item was cued, similar to Exp. 1. A paired sample *t* test revealed a significant difference between mean pupil size when dark versus bright items were cued [*t*(22) = 2.1, *P* = 0.048]. As in Exp. 1, darker items elicited a larger mean pupil size (Fig. 2*E*).

In sum, the findings replicated the top-down modulation of the pupil response according to the brightness of the cued item. Remarkably, the effect occurred even though brightness was neither relevant for orienting attention, which instead was based on item location, nor for the behavioral report of grating orientation. Brightness was merely an additional incidental feature of the memoranda, suggesting some degree of integration among the features constituting cued items in working memory.

### Exp. 3: Pupillary Response According to Dynamic Shifts of Attention to Items of Different Brightness.

**Fig. 3.**
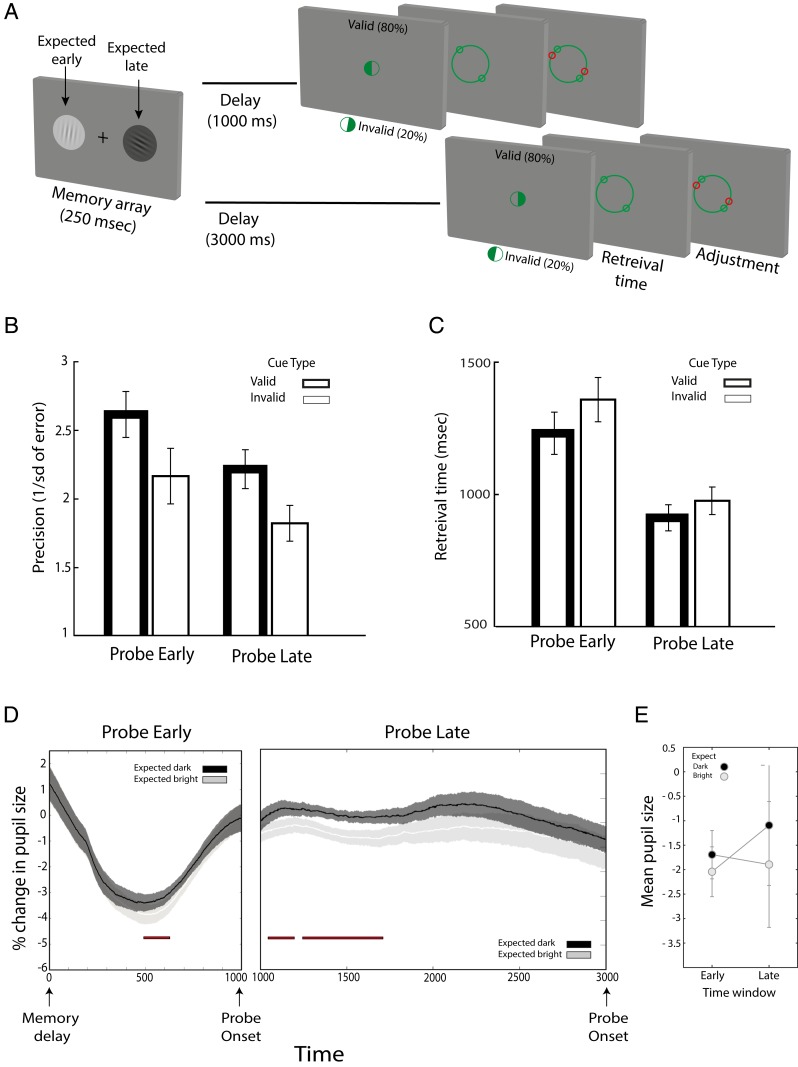
Exp. 3 task schematic (*A*), mean precision (*B*) as a function of the probe time (early vs. late) and probe type (valid vs. invalid), and (*C*) mean retrieval time as a function of probe time (early vs. late) and probe type (valid vs. invalid). (*D*) The influence of temporal validity on pupil diameter. Comparisons of these traces with each other are shown. Darker gratings held in focus of attention elicited a larger change in pupil size compared to brighter gratings in both early and late probe times. The shaded area indicates the SE within subjects. (*E*) Mean pupil size for early vs. late time windows for trials in which either the dark or the bright item was expected to be probed. Error bars indicate SEM, calculated across participants.

In Exp. 3, we tested whether top-down modulation of pupil responses can be flexibly updated as attention moves between items of different brightness in working memory (Fig. 3*A*). We manipulated temporal expectations to vary when participants should prioritize the item on the left vs. right of the remembered array. The time at which the probe appeared indicated the likely item to be probed. For a given set of participants, if the probe appeared early (after 1 s), the left item would most likely be probed (80% validity). If the probe only appeared late (after 3 s), the right item would most likely be probed (80% validity). The spatiotemporal contingencies were reversed for the remaining participants (right then left). The task design, adapted from van Ede et al. (8), allowed us to explore whether participants dynamically shift attention based on the learned spatiotemporal regularities of item relevance and whether pupil responses are similarly dynamically modulated as items of different brightness enter and leave the focus of attention. No extrinsic attention-orienting cues are used in this design, enabling us to test for changes in pupil size in the absence of any auditory cues. As in Exp. 2, item brightness was irrelevant, and only incidental to the task.

#### Behavioral measures.

Repeated-measures ANOVAs tested for within-subject effects of when items were probed (early vs. late probe time) and the validity of temporal expectation (valid vs. invalid) (Fig. 3*B*; see *SI Appendix*, Fig. S1 for mixture modeling of response error).

Precision measures (Fig. 3*B*) were significantly better for items probed early vs. late [*F*(1, 22) = 20.8, *P* < 0.001, η^2^_p_ = 0.49] and for items appearing at the expected interval [*F*(1, 22) = 15.03, *P* = 0.001, η^2^_p_ = 0.41]. The 2 factors did not interact [*F*(1, 22) = 0.9, *P* = 0.7, η^2^_p_ = 0.004]. Retrieval times (Fig. 3*C*) revealed a similar pattern of benefits, with faster responses for items probed early [*F*(1, 22) = 38, *P* < 0.001, η^2^_p_ = 0.63] and for items probed at the expected interval [*F*(1, 22) = 6.8, *P* = 0.016, η^2^_p_ = 0.24]. Again there was no significant interaction between the 2 factors [*F*(1, 22) = 1.1, *P* = 0.3, η^2^_p_ = 0.05].

Brightness could not be added as a factor due to the small number of trials once this factor was also included. However, to examine the effect of brightness of memory performance, we performed 2 separate ANOVAs with within-subject effects of when items were probed (early vs. late) and the brightness of the probed item (dark vs. bright). There was no main effect of brightness on precision [*F*(1, 22) = 1.8, *P* = 0.2, η^2^_p_ = 0.04] or retrieval times [*F*(1, 22) = 0.05, *P* = 0.8, η^2^_p_ = 0.005] and no interaction between brightness and time in which memory was probed for both variables [precision: *F*(1, 22) = 3.9, *P* = 0.06, η^2^_p_ = 0.11; retrieval times: *F*(1, 22) = 0.75, *P* = 0.4, η^2^_p_ = 0.08].

#### Pupil traces.

To analyze pupillary responses, we reorganized the trials based on the brightness of the most relevant item at each time segment (irrespective of the expected side). The first second of all 600 trials were sorted into 2 conditions depending on whether the bright or dark item was at the relevant side. The later interval was analyzed only for trials in which the probe appeared late (300 trials). Trials were divided into those in which the bright or dark item came to occupy the focus of attention. Because traces were analyzed during the anticipation period, it was not necessary to take the validity of the cue into account (see *SI Appendix*, Fig. S3 for pupil size trace for early versus late probed trials separately). The whole memory delay period was taken as the time window of interest, considering that in this experiment, at the time of encoding, 1 item was already prioritized.

Modulations of pupil size were observed during both the early and late part of the delay according to the brightness of the item that should be in the focus of attention (Fig. 3*D*). During the early part of the delay interval, the difference was significant from 645 to 824 ms of the delay period. As in the previous experiments, pupil size was larger when dark items were attended. If no probe appeared, attention shifted to the other item. During this late part of the delay, pupil size also changed, becoming significantly different between 1,071 to 2,255 ms. As before, pupils were relatively dilated when the darker items became attended.

In a complementary analysis, we measured mean pupil size in 2 time windows of interest—early (500 to 1,000 ms) and late (1,500 to 3,000)—to compare responses in trials during which bright stimuli were probed early and dark stimuli were probed late vs. trials with the reverse expectations (dark early, bright late). A repeated-measures ANOVA tested for the brightness of the expected item (dark vs. bright) and time window (early vs. late). Mean pupil size was significantly larger for darker items [*F*(1, 23) = 19.6, *P* < 0.001, η^2^_p_ = 0.47], with no effect of expected time [*F*(1, 23) = 1.8, *P* = 0.2, η^2^_p_ = 0.07] or an interaction between the 2 factors [*F*(1, 23) = 0.15, *P* = 0.7, η^2^_p_ = 0.007], confirming that selecting darker gratings result in larger pupil size, regardless of time interval.

Control analysis confirmed that there were no differences in pupillary responses related to the spatial location of the item attended during the early or late portion of the delay period (minimum *P* = 0.07).

### Memory Error Is Predicted by the Degree of Pupillary Modulation.

In order to examine the relationship between behavioral performance and pupil size within participants, we looked at changes in trial-by-trial normalized measures of error (in degrees) and mean pupil size trace during the last 1,000 ms leading to the probe. In Exps. 1 and 2, the mean of the pupil trace was calculated from 1,000 ms after the cue until the onset of the probe; for Exp. 3 the whole memory delay until the early probe was used. Because error was used as the performance measure, a negative relationship is predicted between performance and pupil size for trials in which the darker item was cued, and a positive relationship is predicted for trials in which the lighter item was cued. A mixed-effects ANOVA with item brightness as within-subject factor and experiment as between-subject factor was conducted on the within-participant correlation coefficients (*SI Appendix*, Fig. S4).

There was a significant difference in mean correlation coefficients between trials in which the bright item versus the dark item was cued across the 3 experiments [main effect of brightness: *F*(1, 65) = 9.69, *P* = 0.003, η^2^_p_ = 0.113] (*SI Appendix*, Fig. S4). There was no significant effect of experiment [*F*(2, 65) = 0.006, *P* = 0.99, η^2^_p_ = 0] or interaction between experiment and brightness [*F*(2, 65) = 0.017, *P* = 0.98, η^2^_p_ = 01]. A similar pattern of modulation occurred for the late-probe condition in Exp. 3. These results indicate that memory is improved on trials where the pupil reflects the cued item.

## Discussion

We investigated whether attention in visual working memory leads to top-down modulations of the pupillary response, in the absence of any visual input. In our first experiment, we observed that prioritizing the dark memory item elicited a larger pupil response compared to prioritizing the bright item, demonstrating that pupil size reflected the item in focus of attention during a blank working-memory delay. In 2 follow-up experiments we went a step further and showed the same pattern of pupil-size modulation when the brightness of the items was entirely task-irrelevant. In addition to providing important replication of our initial finding, pupil modulation according to an incidental feature of an attended item suggests at least some degree of integration of features in working-memory representations. As a corollary, it also highlighted the ability to track the item in the focus of attention via the pupil response. Our findings from Exp. 3 replicate a recent study showing that working-memory memoranda can go in and out of the focus of attention according to dynamically evolving spatiotemporal probabilities of different items being probed (8), and show that the pupil response adjusts dynamically, according to the brightness of the attended item at different intervals, in the absence of an external cue.

Thus, across our 3 experiments, we gathered the first set of clear evidence substantiating the proposal that pupillary responses adjust according to the brightness of items in working memory. Unlike previous studies addressing similar issues (22, 23, 25), our study carefully excludes any possible contamination by presentation or anticipation of visual stimuli of different brightness, in order to rule out explanations based on perceptual attention mechanisms related to selective encoding or anticipation. Our striking observations suggest that attention in working memory draws on similar sensory-orienting mechanisms as external attention (16, 17) to prioritize items.

The relationship between performance error and pupil dilation was significantly different according to the item brightness across all 3 experiments. In other words, smaller errors (better memory precision) were associated with larger pupil sizes for dark items (negative relation) and with smaller pupil sizes for bright items (positive relation). These results are suggestive of a functional link between pupil size and prioritization of the item to be probed. The nature and direction of the relation remains to be determined.

Interestingly, in contrast to pupil-size modulation during attention to anticipated or perceptually available sensory stimuli, there is no obvious adaptive function to adjusting pupil size during attention in working memory, as in our current study. The eyes are not required to access items that exist only as mental representations. Instead, the finding suggests sensory recruitment during working memory, in which neural systems that process the sensory attributes of items during perception also contribute to their maintenance in working memory (28–30). We extend that notion by showing that sensory recruitment can occur all of the way to the peripheral sensory organ, the eye. Extending previous studies of attention in working memory (8–10, 21, 31, 32), we further show that sensory recruitment of the pupillary response can be modulated flexibly according to prioritization and selection of memoranda of different brightness during working-memory delays. Previous studies examining sensory recruitment during working memory have shown that activity in sensory areas reflects primarily the item that is prioritized in working memory, making such items readily decodable in these regions (13, 19, 21). By extension, we showed that attended items can be tracked via the earliest markers of sensory processing, the pupillary light response.

A possible neural mechanism supporting the relationship between attention and pupillary responses is through the involvement the superior colliculus (SC) (33). The SC has been linked to both overt and covert shifts of attention, and proposed to participate in working memory, attention and eye movements (34). The SC receives input from the frontal eye field (35–38), which is linked to both shifts of attention and the pupillary light response (39, 40). Although the exact neural underpinnings remain unknown, our findings may point to an overlap in mechanisms supporting the link between the pupillary response and attentional shifts in perception and working memory.

Our conjecture is that pupil modulation in working memory is a manifestation of sensory recruitment and serves no specific adaptive function. It occurs as a vestige of repurposing basic sensory-motor mechanisms in the service of higher-order cognitive functions, as observed in the strong relationship between spatial movements and spatial attention (41–43). However, whether the pupillary response plays an active functional role during the retention of items in working memory remains to be tested. It is possible, for example, that areas exhibiting sensory recruitment have mutually reinforcing activity, so that pupil modulation can contribute to strengthening the sensory quality of memoranda. Future studies that modulate pupil size in relation to brightness levels of attended vs. ignored stimuli in working memory could test this intriguing possibility.

Remarkably, in 2 of our experiments, modulation of the pupillary response occurred even when the brightness of the working-memory items was completely task-irrelevant. Brightness was neither used to orient attention nor probed at recall. Hence, this incidental feature could have been ignored. Instead, our findings point to the preservation of this irrelevant feature in the memoranda, suggesting some degree of automatic feature integration in working memory. Some features, such as spatial location and temporal order have been observed to be preserved and retrieved in working-memory tasks even when not directly task-relevant (30, 44). Such findings suggest that the spatial and temporal dimensions may be essential for organizing mental representations in working memory. Brightness is unlikely to have a similarly important anchoring role, but our observations clearly indicate that information about low-level stimulus properties, such as brightness, are integrated into the representation of the prioritized items in working memory, in line with behavioral findings that report interference from task-irrelevant features in working memory (12, 45–51). Together these findings suggest that recall involves reactivating the perceptual representation of a whole object (26, 52)

To conclude, the present set of experiments demonstrate that the pupils can reflect the brightness of the prioritized memorandum even when brightness is task-irrelevant, and when no differential light is expected on the retina. These findings highlight the repurposing of sensory mechanisms to guide adaptive behavior based on attention functions and memory traces, all of the way down to the pupils. Attentional prioritization in working memory is highly dynamic and the results presented here highlight a promising avenue for using the pupil light response as a means to track these dynamic shifts of attention during working memory. Whether such pupil dilation in the absence of any differential light stimulation or expectation plays a functional role in these working-memory representations remains to be tested.

## Materials and Methods

All experiments were approved by the local ethics committee (Central University Research Ethics Committee). Participants were different for each experiment and provided written informed consent before participation. Sample sizes were determined based on previous studies on working-memory prioritization and on examining changes in pupil response during tasks of attention (8, 17). All participants had normal or corrected-to-normal visual acuity and normal color vision. They were compensated for their time at an hourly rate of £10.

### Experiment 1.

#### Participants.

Twenty-five healthy volunteers (6 male), with mean age of 24.5 y (range 22 to 29 y) participated in Exp. 1. Three participants were excluded from analysis due to eye-tracking error during testing, leaving 22 volunteers (5 male, mean age of 24 y) in the final sample.

#### Experimental design and procedures.

The experimental task was programmed and controlled using Matlab. Stimuli were presented on a 24-in Viewsonic monitor with a spatial resolution of 1,280 × 720 pixels and 100-Hz refresh rate. Participants viewed the monitor using a head rest at a distance of ∼90 cm from the eye. Eye movements and pupil size were recorded by an Eyelink 1,000-Hz infra-red eye at 1,000-Hz sampling rate. Participants were required to maintain fixation throughout the trial. Auditory cues were presented via headphones at a fixed loudness value.

A schematic of the experimental design is illustrated in Fig. 1*A*. Participants performed a visual working-memory task that required them to keep in mind, for a short duration of time, the orientations of 2 gratings presented briefly (250 ms) to the right and left of the fixation cross (4° eccentricity). The gratings had a diameter of 2° visual angle and a spatial frequency of ∼2 cycles per degree. The background of one of the gratings was dark gray while the other was a bright gray, 50% darker or brighter than the gray background screen respectively. The orientations of the 2 gratings were randomly selected for each trial and independent of one another.

Following a short delay (500 ms), 1 of the 3 distinguishable auditory cues was presented for 100 ms (cues were composite sounds chosen to be readily discriminable). On two-thirds of the trials, the cue was valid, and indicated with 100% certainty the item that would later be probed (sound 1 cued the darker grating, while sound 2 cued the brighter grating). In the remaining third of the trials, the cue was uninformative of the upcoming probe (neutral cue), and hence participants had to remember both items. The cue was followed by a blank memory delay of 2,000 ms before the appearance of the probe.

The probe consisted of a filled green circle presented at screen center (0.5° diameter). For trials with valid, informative cues, a constant midbrightness green probe prompted participants to reproduce the orientation of the retrocued grating. The probe was uniform and carried no information regarding the grating participants had to recall. This ensured that participants paid attention to the retrocue sound. In the neutral retrocue condition, the color of the circle indicated which item should be retrieved for reporting the grating orientation: A light green probe (50% lighter than the neutral green) prompted report based on the bright grating and the dark green circle (50% darker than the neutral green) prompted report based on the dark grating.

When the probe appeared, participants indicated when they were ready to report the orientation of the relevant grating by using a mouse click. Response times between appearance of the probe and the mouse click were taken as a measure of the retrieval time to report the memorandum. The mouse click lead to the appearance of a report prompt. This consisted of a green circle, in the same color as the probe (2° diameter) with 2 “handles” (Fig. 1*A*). The handles were placed on opposite sides to indicate the endpoints of an oriented line. Participants used the mouse to adjust the handles to match the orientation of the probed item. The initial location of the handles was selected randomly. Feedback was provided immediately after response.

Participants completed 7 blocks of 60 trials each (420 trials in total) after becoming familiarized with task procedures in a practice block. Participants learned the association between the sounds and cue identity prior to the experiment and the sounds were randomly assigned to a cue condition in each participant.

#### Pupil analysis.

To examine the pupil response to the item in memory, we looked at pupil size traces, 500 ms after the offset of the cue sound. Trials in which participants did not maintain fixation and made saccades to the memory items were excluded from analysis (<1% of overall trials; see *SI Appendix* for mean percentage of trials used per condition and for each experiment) (*SI Appendix*, Table S1). To compare pupil size traces between cued and uncued dark and bright gratings, we used permutation testing to correct for multiple comparisons. First, standard preprocessing was performed (for similar procedure, see ref. 24). For each trial, data were epoched to obtain pupil size for the time window of interest (from cue onset to probe onset), blinks were interpolated, and a baseline period (200 ms from onset of the auditory cue) was subtracted; therefore the first 200 ms (including cue duration of 100 ms) were used as the baseline for the pupil size trace. Clean and baselined epochs were averaged according to trial conditions to calculate an average pupil size trace for each condition for each participant. These mean traces were randomly permutated, independently for each participant, to give a new dataset with shuffled condition labels. For each random permutation, the *t*-statistics for the contrast of interest was computed at each time point along the trace for the time window of interest. The maximum value of this *t*-statistic over all time points within the window of interest on trace was calculated, and the “maximum” was computed for 5,000 permutations. This gives a null distribution of the maximum *t*-statistics. A *P* value could then be obtained by comparing the *t*-statistics of the original unshuffled data at each time-point with this reference distribution. This *P* value thus controls for the family-wise error rate.

### Experiment 2.

#### Participants.

Twenty-five healthy volunteers participated in Exp. 2 (7 male, mean age: 24 y, range 21 to 29 y). Two participants were excluded from analysis due to eye-tracking error during testing or unusable eye-tracking data, leaving 23 volunteers (7 male, mean age of 24 y) in the final sample.

#### Experimental design and procedures.

Exp. 2 was identical to Exp. 1 in design and procedures, except for the meanings of the cues and the appearance of the probe stimuli. In this experiment, the auditory cues indicated the location, and not the brightness, of the item to be reported. Valid spatial auditory retrocues occurred on two-thirds of trials, indicating, with 100% validity, whether the left or right item would be probed. In the remaining third of the trials, the auditory retrocue provided no information as to the location of the item to be probed (neutral retrocue). For valid retrocue trials, the probe stimulus was identical to that used in Exp. 1. In neutral-cue trials, the probe indicated the spatial location of the item to be reported by having the probe circle half-filled (Fig. 2*A*). For example, the filled right side of the circle indicated that the orientation should be reported for the item on the right side of the remembered array. The brightness of the gratings was irrelevant and orthogonal to the cued side.

Pupil size trace analysis was identical to that described in Exp. 1.

### Experiment 3.

#### Participants.

Twenty-eight healthy volunteers participated in Exp. 3 (12 male, mean age: 25.6 y, range 20 to 30 y). All participants had normal or corrected-to-normal visual acuity and normal color vision. Five participants were excluded from analysis due to eye-tracking error during testing or due to lack of good quality eye-tracking, leaving 23 volunteers (9 male, mean age of 24.7 y) in the final sample.

#### Experimental design and procedures.

Exp. 3 built on Exp. 2 by including learned spatiotemporal expectations that indicated the likely location of the item to be probed after a short (1,000 ms) or long (3,000 ms) memory delay. The design was modeled on the study be van Ede et al. (8). Participants learned that items on a given side (e.g., left) were likely to be probed early (1,000 ms) on the majority (80%) of trials and that items on the other side (e.g., right) were likely to be probed late. The side that was more likely to be probed early was balanced across participants. No auditory retrocues were used and no marker indicated the end of the first interval in which an item could be probed. Instead, only the tracking of the passage of time indicated which item should be prioritized.

The probe stimulus consisted of a half-filled circle presented at screen center. The filled half indicated the location of the item to be reported. A report prompt followed, and participants adjusted its handles to match the orientation of the probed grating. Feedback was provided immediately after response.

Participants completed 10 blocks of 60 trials each (600 trials in total, 480 validly cued trials) after becoming familiarized with task procedures in a practice block. Participants were explicitly informed about the association between probe time and location of the relevant grating prior to the experiment. They gained experience with the association by completing practice blocks.

Pupil size trace analysis was identical to that described in Exp. 1, except that for each trial, data were epoched from memory delay onset to probe onset, using a baseline period of 200 ms (from memory delay onset).

### Data Availability.

All data discussed in the paper will be made available upon request.

## Supplementary Material

Supplementary File
