# Supplementary material for: Modulation of the pupillary response by the content of visual working memory
Source: Proc Natl Acad Sci U S A. 2019 Oct 21;116(45):22802–10. doi: 10.1073/pnas.1909959116 (PMC6842592; doi:10.1073/pnas.1909959116)
Supplement: Supplementary File [file pnas.1909959116.sapp.pdf]

PNAS
www.pnas.org

Supplementary Information for

Adjusting the aperture of the mind's eye: modulation of the pupillary response by the content of visual working memory

Nahid Zokaei, Alexander G Board, Sanjay G Manohar & Anna Christina Nobre

Nahid Zokaei
Email: nahid.zokaei@psy.ox.ac.uk

This PDF file includes:

Table S1
Supplementary text
Figures S1 to S4
SI References

www.pnas.org/cgi/doi/10.1073/pnas.1909959116

Average number of trials in the pupil size analysis per experiment

Table S1. Mean percentage of trials as well as the range of number of trials included in the final analysis of the pupil size trace per experiment and in each condition.

	**Maximum number of trials per condition**	**Dark item cued** Mean % of usable trials, (sdev); range (n of trials)	**Bright item cued** Mean % of usable trials, (sdev); range (n of trials)
Exp 1	140	99.4 (2.2); 132-140	99.2 (1.7); 133-140
Exp 2	140	99.1 (1.9); 129-140	99 (2.1); 130-140

	**Maximum number of trials per condition**	**Dark Early** Mean % of usable trials, (sdev); range (n of trials)	**Bright Early** Mean % of usable trials, (sdev); range (n of trials)	**Dark Late** Mean % of usable trials, (sdev); range (n of trials)	**Bright Late** Mean % of usable trials, (sdev); range (n of trials)
Exp 3	150	99.2 (1.95); 135-149	99 (2.09); 133-149	99.3 (1.7); 137-148	99.5 (1.6); 141-149

Supplementary Information Text

Mixture modelling of behavioural data

We applied mixture modelling [1] to behavioural data across the 3 experiments in order to separate the sources of error contributing to performance as measured by recall precision. The Concentration parameter captures errors arising from increased variability in the quality of the memory for the orientation of the probed grating, with higher values corresponding to lower variability. Alternatively, random guessing occurs when participants cannot recall the orientation of the probed item and hence make a guess instead. Finally, swap errors (or misbinding) occurs when participants incorrectly report the orientation of the non-probed item in memory, hence swapping the features of items in working memory.

Modelling results (Figure S1) showed that the effects of cue validity were expressed through the Concentration parameter across all three experiments (main effect of validity – Exp01: $F(1,21)= 4.49$, $p=0.046$, $\eta^2_p=0.14$; Exp02: $F(1,21)= 5.\ 9$, $p=0.023$, $\eta^2_p=0.21$; Exp03: $F(1,21)= 6.46$, $p=0.019$, $\eta^2_p=0.23$). There was no effect of validity or brightness on any of the other model estimates.

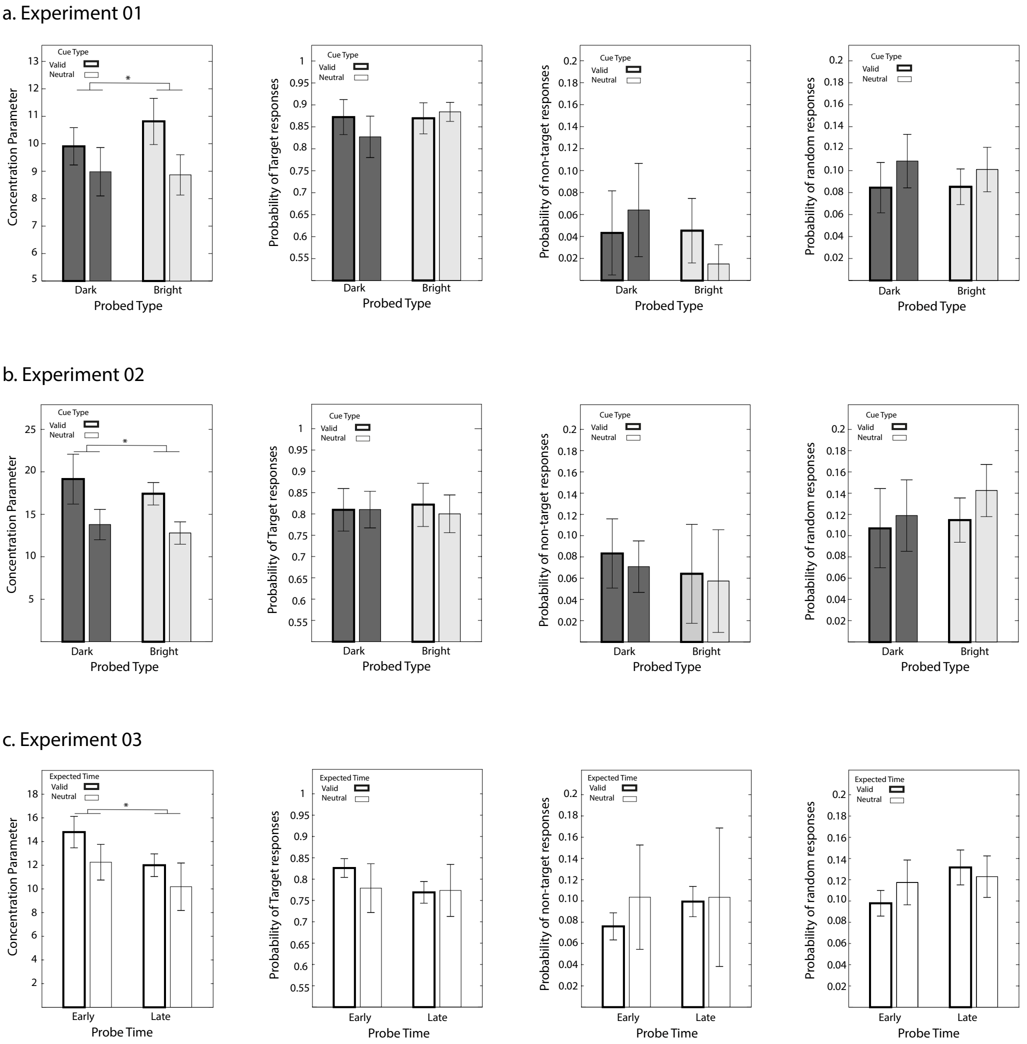

Figure S1. Model estimates of concentration parameter, probability of target responses, swapping (non-target responses), and random responses (guessing) for the 3 experiments.

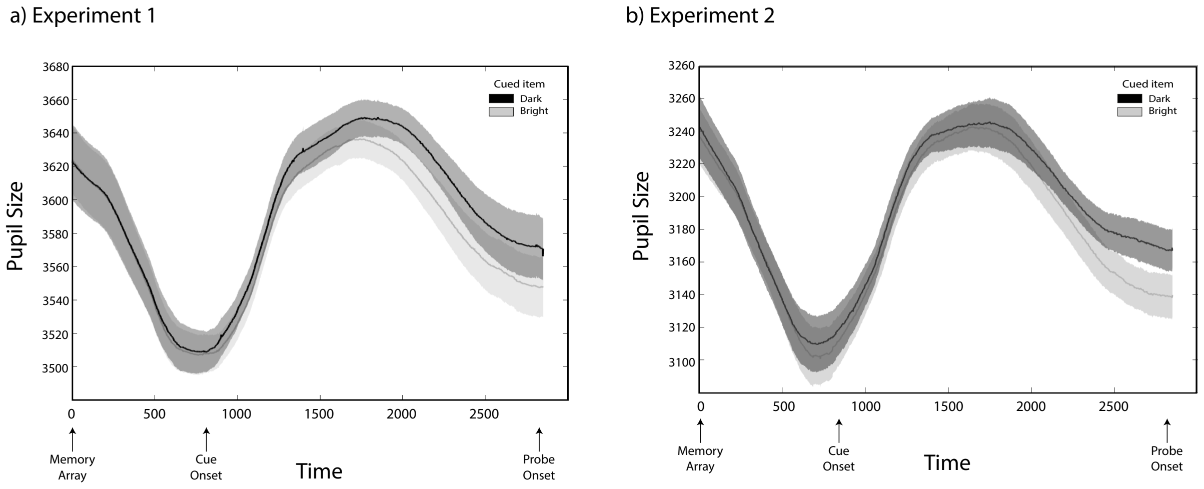

Figure S2. Raw pupil traces for Experiments 1 and 2 from memory array onset until probe for dark vs. bright cued gratings in (a) Experiment 1 and (b) Experiment 2.

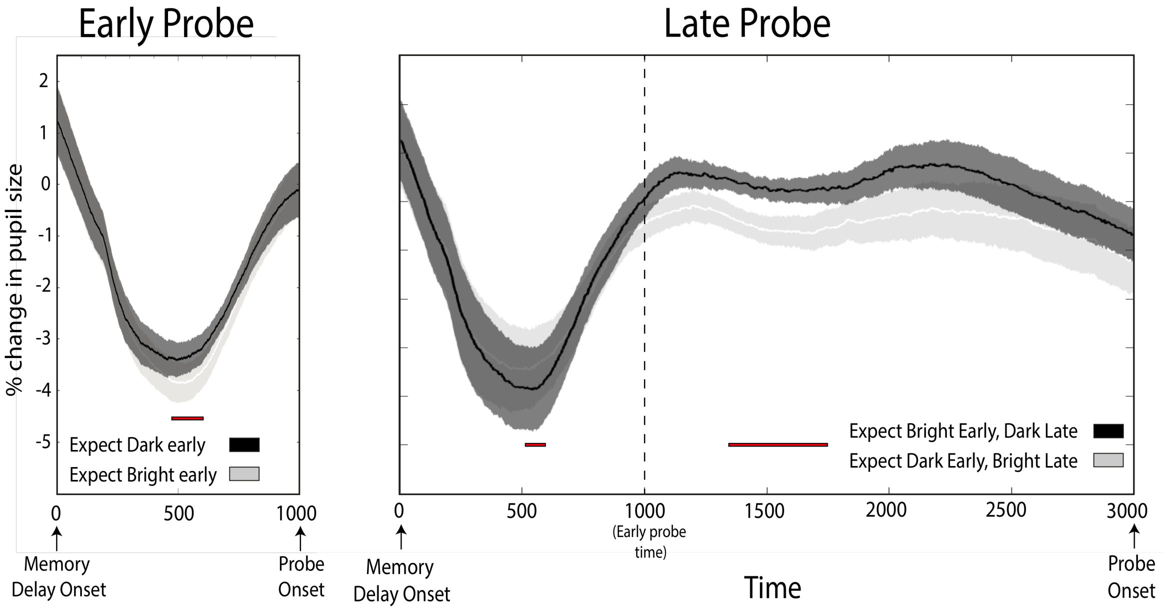

Figure S3. Pupil traces for trials in which participants were probed either early (left hand panel) or late (right hand panel), sorted by the brightness of the item in focus of attention at the time of the probe. Darker gratings held in focus of attention elicited a larger pupil size compared to brighter gratings. Importantly, for trials in which there is a shift in the brightness of the item in focus of attention, that is trials in which the probe appeared later in the trial (right hand side), there is a cross-over of the pupil trace tracking the brightness of the expected grating. Error bars indicate SEM, calculated across participants (red lines indicate significant segments).

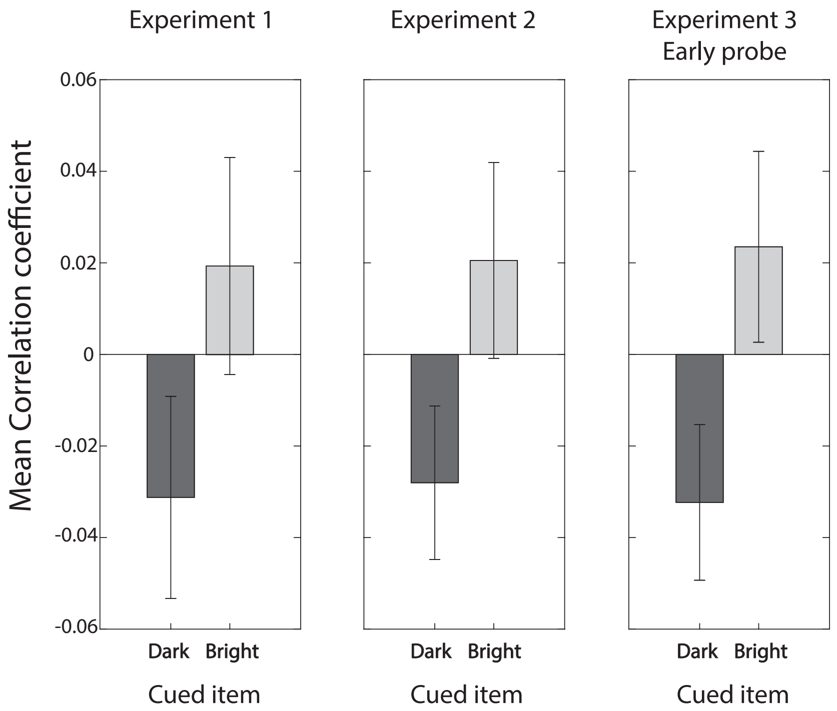

Figure S4. The relationship between trial-by-trial error and pupil size across 3 experiments. Mean correlation coefficients for the relationship between performance error and mean pupil size during the 1000 ms preceding the probe for Experiments 1, 2, and the early probe. In Experiment 3 the whole memory delay until the early probe was used. Error was calculated as the angular difference between reported orientation and the true orientation of the grating.

References

[1] Bays PM, Catalao RFG, Husain M. The precision of visual working memory is set by allocation of a shared resource. Journal of Vision 2009;9. doi:10.1167/9.10.7.
